# *Trypanosoma* Infection Favors *Brucella* Elimination *via* IL-12/IFNγ-Dependent Pathways

**DOI:** 10.3389/fimmu.2017.00903

**Published:** 2017-07-31

**Authors:** Arnaud Machelart, Margaux Van Vyve, Georges Potemberg, Aurore Demars, Carl De Trez, Hermann Giresse Tima, Gilles Vanwalleghem, Marta Romano, Carine Truyens, Jean-Jacques Letesson, Eric Muraille

**Affiliations:** ^1^Unité de Recherche en Biologie des Microorganismes, Laboratoire d’Immunologie et de Microbiologie, NARILIS, Université de Namur, Namur, Belgium; ^2^Department of Molecular and Cellular Interactions, Vlaams Interuniversitair Instituut voor Biotechnologie, Vrije Universiteit Brussel, Brussels, Belgium; ^3^Service Immunology, Scientific Institute for Public Health (WIV-ISP), Brussels, Belgium; ^4^Laboratory of Molecular Parasitology, IBMM, Université Libre de Bruxelles (ULB), Gosselies, Belgium; ^5^Laboratoire de Parasitologie, Faculté de Médecine, Université Libre de Bruxelles (ULB), Bruxelles, Belgium

**Keywords:** infection control, *Trypanosoma cruzi*, *Trypanosoma brucei brucei*, *Brucella melitensis*, *Brucella abortus*, brucellosis, *Mycobacterium tuberculosis*

## Abstract

This study develops an original co-infection model in mice using *Brucella melitensis*, the most frequent cause of human brucellosis, and *Trypanosoma brucei*, the agent of African trypanosomiasis. Although the immunosuppressive effects of *T. brucei* in natural hosts and mice models are well established, we observed that the injection of *T. brucei* in mice chronically infected with *B. melitensis* induces a drastic reduction in the number of *B. melitensis* in the spleen, the main reservoir of the infection. Similar results are obtained with *Brucella abortus*- and *Brucella suis*-infected mice and *B. melitensis*-infected mice co-infected with *Trypanosoma cruzi*, demonstrating that this phenomenon is not due to antigenic cross-reactivity. Comparison of co-infected wild-type and genetically deficient mice showed that *Brucella* elimination required functional IL-12p35/IFNγ signaling pathways and the presence of CD4^+^ T cells. However, the impact of wild type and an attenuated mutant of *T. brucei* on *B. melitensis* were similar, suggesting that a chronic intense inflammatory reaction is not required to eliminate *B. melitensis*. Finally, we also tested the impact of *T. brucei* infection on the course of *Mycobacterium tuberculosis* infection. Although *T. brucei* strongly increases the frequency of IFNγ^+^CD4^+^ T cells, it does not ameliorate the control of *M. tuberculosis* infection, suggesting that it is not controlled by the same effector mechanisms as *Brucella*. Thus, whereas *T. brucei* infections are commonly viewed as immunosuppressive and pathogenic, our data suggest that these parasites can specifically affect the immune control of *Brucella* infection, with benefits for the host.

## Introduction

In natural populations, individual resistance to infection is remarkably diverse. This has been linked to many factors [reviewed in Ref. (1)]. Among them, persistent infection can enhance the ability to control unrelated pathogens, a phenomenon termed the “Mackaness effect” in reference to the seminal work of Mackaness (2, 3) demonstrating cross-protection between *L. monocytogenes, Brucella abortus*, and *Mycobacterium tuberculosis* infections in mice. Since this pioneering work, numerous examples of the Mackaness effect have been reported. For example, Herpes virus infection can provide beneficial protection against *L. monocytogenes* and *Yersinia pestis* (4). On the contrary, the lethal synergism between *Influenza virus* and certain bacteria, particularly *Streptococcus pneumoniae*, is well documented (5). As multiple unrelated infections in the same host are probably more common than single infections, it would be interesting to better understand the mechanisms underlying these cross-protections or cross-aggravations.

*Brucellae* (alpha-proteobacteria) are facultative intracellular Gram-negative coccobacilli that infect mammals and cause brucellosis. Human brucellosis is a zoonotic infection transmitted mainly through ingestion and inhalation (6). Without prolonged antibiotic treatment it causes a severe and debilitating chronic disease (7, 8). Despite significant progress, the incidence of human brucellosis remains very high in endemic areas, such as North Africa, the Mediterranean basin, and South America (9), and is considered to be largely underestimated (10). There is still no available safe and protective vaccine for humans (11, 12). *Brucella melitensis* is the most frequent cause of human brucellosis (8). Whole-body imaging of mice infected with high doses of bioluminescent *B. melitensis* has confirmed that the mouse infection model parallels human infection and identified major sites of bacterial growth and persistence, such as the spleen (13). Although the precise mechanisms of protective immunity against *Brucella* remain largely unknown, the role of IFNγ-producing CD4^+^ T cells (Th1) in the control of *Brucella* growth in the spleen of infected mice is well established (14–16).

*Trypanosoma brucei* is a strictly extracellular parasitic protozoan hemoflagellate that causes African trypanosomiasis, also known as sleeping sickness in humans and nagana in animals. The mammalian bloodstream forms of *T. brucei* are remarkable for their variant surface glycoprotein coats that undergo antigenic variation, thus enabling persistent escape from host adaptive immunity and chronic host infection [for a review, see Ref. (17)]. Wild-type C57BL/6 mice infected with *T. brucei* were characterized by an initial parasitemic surge inducing an intense IFNγ inflammatory response followed by subsequent cyclic parasitemic waves of smaller amplitude than the first peak. During chronic infection, *T. brucei* causes immunosuppression by various mechanisms. In particular, *T. brucei* induces the loss of various B-cell populations by apoptosis and thus abrogates the vaccine-induced protective response to a non-related pathogen (18). It also suppress the T-cell response by IFNγ/nitric oxide-dependent and -independent pathways (19, 20).

Based on bibliographic data, we hypothesize that infection with *T. brucei* may affect the control of primary *B. melitensis* infection and the development of protective memory. To test these hypotheses, we develop an original co-infection experimental model. *B. melitensis*-infected mice were infected with *T. brucei* at early and later time points. Surprisingly, co-infection induced a rapid and drastic reduction in the number of *B. melitensis* in the spleen and often its complete elimination. This phenomenon appeared to be dependent on IFNγ and CD4^+^ T cells.

## Materials and Methods

### Ethics Statement

The procedures used in this study and the handling of the mice complied with current European legislation (directive 86/609/EEC) and the corresponding Belgian law “Arrêté royal relatif à la protection des animaux d’expérience du 6 avril 2010 publié le 14 mai 2010.” The Animal Welfare Committee of the Université de Namur (UNamur, Belgium) reviewed and approved the complete protocol (Permit Number: 12-188).

### Mice and Reagents

Wild-type C57BL/6 mice were acquired from Harlan (Bicester, UK). IL1R^−/−^ C57BL/6, CD3ε^−/−^ C57BL/6, and TCR-δ^−/−^ C57BL/6 mice were purchased from The Jackson Laboratory (Bar Harbor, ME, USA). IL-12p35^−/−^ C57BL/6 mice (21) were acquired from Dr. B. Ryffel (University of Orleans, France). TAP1^−/−^ C57BL/6 mice (22) and MHCII^−/−^ C57BL/6 mice (23) were acquired from Jörg Reimann (University of Ulm, Ulm, Germany). CD11c-DTR C57BL/6 mice were obtained from Dr. G. Holdenhove (Université Libre de Bruxelles, Belgium) and injected intraperitoneally (i.p.) with 500 ng of diphtheria toxin (DT) (Sigma) in PBS or with PBS alone (control). All wild-type and deficient mice used in this study were bred in the animal facility of the Gosselies campus of the Université Libre de Bruxelles (ULB, Belgium).

### *Brucella* Infection

We used wild-type *B. melitensis* 16M and strains stably expressing a rapidly maturing variant of the red fluorescent protein DsRed (mCherry-Br) (24), the mCherry protein (mCherry-Br), under the control of the strong *Brucella* spp. promoter, PsojA. Construction of the mCherry-Br strains has been described previously in detail (25). We also used *B. abortus* 2308 and *Brucella suis* 1330. All *Brucella* strains were grown in biosafety level III laboratory facilities. Cultures were grown overnight with shaking at 37°C in 2YT medium (Luria–Bertani broth with double quantity of yeast extract) and were washed twice in RPMI 1640 (Gibco Laboratories) (3,500 × *g*, 10 min) before inoculation of the mice. The mice were anesthetized with a cocktail of xylazine (9 mg/kg) and ketamine (36 mg/kg) in PBS before being inoculated intranasally (i.n.) with 2 × 10^4^ CFU of *B. melitensis, B. abortus*, and *B. suis* in 30 µl of PBS [described in Ref. (25)]. For the i.p. infection with *B. melitensis*, the mice received 2 × 10^4^ CFU/500 μl of PBS. Control animals were inoculated with the same volume of PBS. The infectious doses were validated by plating serial dilutions of the inocula. The mice were sacrificed at the selected time after infection by cervical dislocation. Immediately after sacrifice, spleen cells were collected for bacterial count and flow cytometry analyses.

### *M. tuberculosis* Infection

C57BL/6 mice were infected with 50–100 CFU of virulent *M. tuberculosis* H37Rv using a nose-only inhalation exposure system (CH Technologies, Inc., Westwood, NJ, USA). The *M. tuberculosis* H37Rv strain used was grown for 2 weeks as a surface pellicle on Sauton medium and stored frozen in aliquots at −80°C and is transformed with the reporter plasmid pSMT1, which expresses the *Vibrio harveyi luxAB* genes under the control of the BCG hsp60 promoter (26). The number of bioluminescent organisms [determined as relative light units (RLU)] in spleen homogenates was determined by a bioluminescence assay with a Modulus luminometer (Turner Biosystems) and 1% *n*-decanal in ethanol as a substrate. Data are expressed as log10 mRLU values per organ per mouse. All *M. tuberculosis* infections were performed in a BSL3 facility at the Scientific Institute of Public Health (WIV-ISP) according to rules established by the ethics committee of the WIV-ISP and CODA-CERVA (permit 060202-02).

### *Trypanosoma* Infection

The pleomorphic AnTat 1.1E (EATRO 1125 stock) *Trypanosoma brucei brucei* (18) and a dominant-negative adenylate cyclase (DNac) mutant (27) were used in this study. *T. brucei* infection is characterized by multi-wave parasitemic development, in which every wave represents a parasite population of different antigenic type. DNac mutant parasitemia is considerably lower and displays no peak of infection (27). The mice were infected by i.p. injection of 5,000 parasites/mouse. Every 2–3 days, the number of parasites present in the blood was estimated using a counting chamber and a light microscope. For *T. brucei* elimination, the mice were treated by i.p. injection of Berenil (diminazene aceturate, 14 mg/kg, Sigma-Aldrich) in 200 µl of distillated water (28). A parasite lysate was obtained by three freeze/thaw cycles as described in Ref. (29).

For *Trypanosoma cruzi*, we used the Tulahuen strain (genotype TcVI). The mice were infected by i.p. injection of 1,000 blood trypomastigotes as previously described (30). Blood parasitemia was evaluated regularly by microscopic examination.

### Antibiotic Treatment

Antibiotic treatment was administered to both immunized and control mice for 2 weeks. The oral treatment was a combination of rifampicin (12 mg/kg) and streptomycin (450 mg/kg) [adapted from Ref. (31)] prepared fresh daily and given in the drinking water. To ensure that the antibiotic treatment was effective, some mice from each group were sacrificed 1 week prior to the challenge, and the colony-forming unit counts were evaluated in the spleen.

### *Brucella* Counting

Spleens were crushed and transferred to PBS/0.1% X-100 Triton (Sigma-Aldrich). We performed successive serial dilutions in RPMI to obtain the most accurate bacterial count and plated them onto 2YT medium. The colony-forming units were counted after 5 days of culture at 37°C.

### Enzyme-Linked Immunosorbent Assay (ELISA)

*Brucella*-specific murine IgM and IgG isotypes were determined by ELISA. Polystyrene plates (Nunc 269620) were coated with heat-killed *B. melitensis* (10^7^ CFU/ml). After incubation overnight at 4°C, the plates were blocked for 2 h at room temperature (RT) with 200 µl of PBS-3.65% casein. The plates were then incubated overnight at 4°C with 50 µl of serial dilutions of the serum in PBS-3.5% casein. The sera of unimmunized mice were used as the negative control. After four washes with PBS, isotype-specific goat anti-mouse horseradish peroxidase conjugates were added (50 μl/well) at appropriate dilutions (hIgM from Sigma; LO-MG2a-9 HRPO). The plates were incubated for 2 h at RT and washed four times in PBS before adding 100 µl of substrate solution (BD OptEiA) to each well. After 10 min of incubation at RT in the dark, the enzyme reaction was stopped by adding 25 μl/well of 2 N H_2_SO_4_, and absorbance was measured at 450 nm.

### Cytofluorometric Analysis

As described previously (16), spleens were harvested, cut into small pieces and incubated for 30 min at 37°C with a mix of DNAse I fraction IX (Sigma-Aldrich) (100 µg/ml) and 1.6 mg/ml of collagenase (400 M and l U/ml). The spleen cells were washed and filtered, and then incubated with saturating doses of purified 2.4G2 (anti-mouse Fc receptor, ATCC) in 200 µl PBS 0.2% BSA 0.02% NaN_3_ (FACS buffer) for 20 min at 4°C to prevent antibody binding to the Fc receptor. Various fluorescent mAb combinations in FACS buffer were used to stain 3–5 × 10^6^ cells. We acquired the following mAbs from BD Biosciences: phycoerythrin (PE)-coupled HL3 (anti-CD11c), FITC-coupled 145-2C11 (anti-CD3ε), FITC-coupled M1/70 (anti-CD11b), PE-coupled RM4-5 (anti-CD4), allophycocyanin (APC)-coupled 1-A/1-E (anti-MHCII), and APC-coupled XMG1.2 (anti-IFNγ). Purified M-19 (rabbit polyclonal IgG anti-NOS2; Santa Cruz Biotechnology) was stained with Alexa Fluor 647 goat anti-rabbit (Molecular Probes). The cells were analyzed on a FACScalibur cytofluorometer. Dead cells and debris were eliminated from the analysis according to size and scatter.

### Statistical Analysis

We used a (Wilcoxon–)Mann–Whitney test provided by the GraphPad Prism software to statistically analyze our results. Each group of deficient mice was compared to the wild-type mice. We also compared each group with each other and displayed the results when required. Values of *p* < 0.05 were considered to represent a significant difference. *, **, and *** denote *p* < 0.05, *p* < 0.01, and *p* < 0.001, respectively.

## Results

### *B. melitensis* Persists in CD11c^+^ Reservoir Cells in the Spleen and Is Able to Resist to Protective Memory Immune Response

The identification of reservoir cells allowing for the persistence of *Brucella in vivo* constitutes a crucial step in our understanding of how *Brucella* escapes the immune system. Following i.n. administration of 2 × 10^4^ CFU of *B. melitensis*, we have shown previously (16) that the bacteria persist in the lungs up to 12 days postinfection and the spleen and liver are colonized starting 5 days postinfection. At 28 days postinfection, *B. melitensis* is only detected in the spleen by CFU analysis. At that time, it is not detected in the lungs, liver, brain, ovaries, heart, thigh muscle, and tissues of the tail (data not shown). Microscopic analysis has demonstrated that during the chronic phase of infection of highly susceptible IL-12p40^−/−^ BALB/c mice, *B. melitensis* resides in specific reservoir cells expressing a particular phenotype (CD11c^+^CD11b^−^CD205^+^arginase^+^) (32). Due to the low number of *B. melitensis* persisting in the spleen of resistant mice (10^3^–10^4^ CFU/spleen, less than 1 infected cell per 10^4^ spleen cells), *in situ* microscopic analysis of the phenotype of the infected cells is not possible. To solve this problem, we chose to use CD11c-DTR C57BL/6 mice that express the DT receptor under the control of the CD11c promoter. As previously reported (33), injection of DT in DTR mice induced the transitory elimination of CD11c^+^ cells.

**Figure 1:**
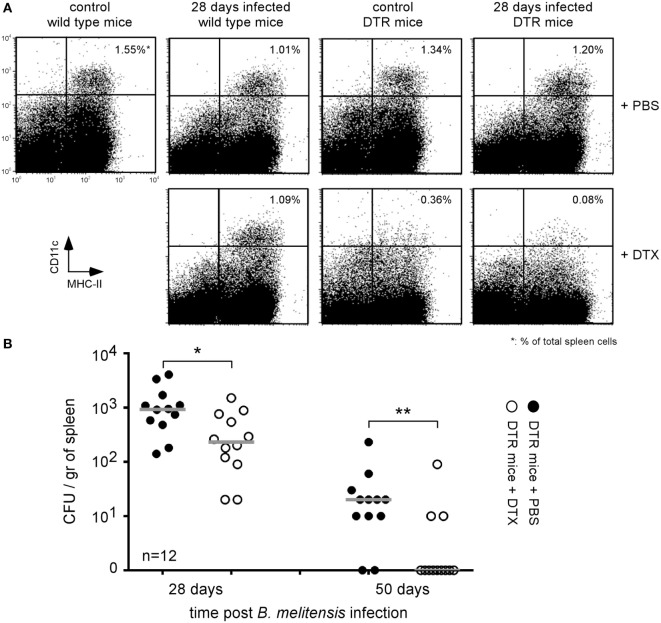
*Brucella* persisted in CD11c^+^ reservoir cells in the spleen of chronically infected wild-type C57BL/6 mice. Wild-type and DTR-CD11c C57BL/6 mice received i.n. 2 × 10^4^ CFU of mCherry-*Brucella melitensis* in PBS or PBS alone (control group). 26 or 48 days later, the mice received 500 ng of diphtheria toxin by i.p. route in 500 µl of PBS or PBS alone. Two days later (28 or 50 days post *Brucella* infection), the mice were sacrificed, and the spleens were harvested. **(A)** Flow cytometry analysis of CD11c and MHCII expression on spleen cells. The data show a representative dot plot for individual mice. **(B)** The data represent the number of colony-forming units per gram of spleen for each group of mice at the indicated time of infection. Gray bars represent the median. *n* denotes the number of mice used for each group (*p < 0.05, **p < 0.01). These results are representative of at least two independent experiments.

To determine if the *B. melitensis* reservoir cells express CD11c, DTR mice infected for 26 or 48 days with 2 × 10^4^ CFU of mCherry-*B. melitensis* received 500 ng of DT in 500 µl of PBS or PBS alone (control group). Two days later, the mice were sacrificed and the spleens were harvested. Flow cytometry analysis showed that DT induced a depletion of CD11c^high^ cells in control and *Brucella*-infected DTR mice but not in wild-type mice (Figure 1A). Elimination of the CD11c^+^ cells in DTR mice led to a drastic reduction in the number of bacteria compared to the mice treated with PBS alone (Figure 1B). Note that, as expected, we observed no significant impact of DT administration on the control of *Brucella* infection in wild-type C57BL/6 mice (Figure S1 in Supplementary Material). These results suggest that *Brucella* persists in CD11c^+^ reservoir cells in wild-type C57BL/6 mice.

**Figure 2:**
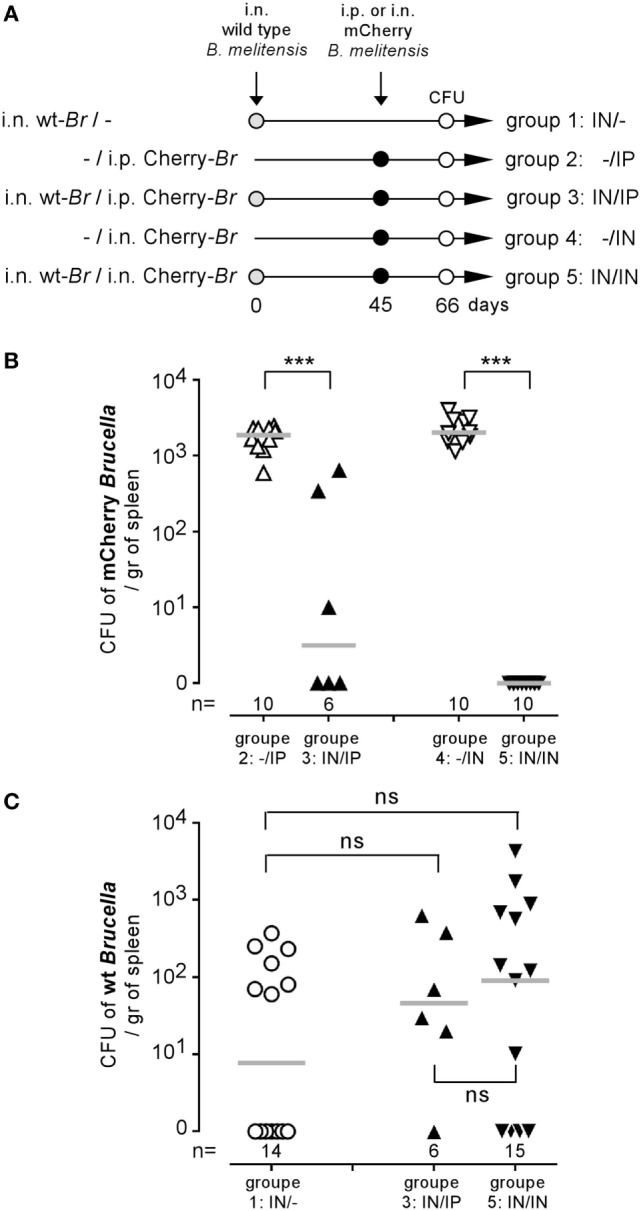
*Brucella* reservoir cells were resistant to the protective memory response. Wild-type C57BL/6 mice were infected i.n. with 2 × 10^4^ CFU of wild-type *Brucella melitensis* and challenged i.p. or i.n. with 2 × 10^4^ CFU of mCherry-*B. melitensis*. The mice were euthanized at the selected time, and the spleen was harvested, as described in panel **(A)**. The data in panels **(B,C)** represent the number of colony-forming units per gram of spleen for each group of mice. Gray bars represent the median. *n* denotes the number of mice used for each group (***p < 0.001). These results are representative of at least two independent experiments.

We have previously shown that i.p. (31) or i.n. (16) *Brucella* infection in wild-type C57BL/6 mice induces a protective memory T cell response able to control and completely eliminate a secondary *Brucella* infection in a majority of mice. To determine whether the activation of memory T cells during a secondary infection is able to eliminate *Brucella* from the first infection persisting in splenic reservoir cells, we compared the CFU levels of non-fluorescent (wild-type) *B. melitensis* and mCherry-*B. melitensis* in the spleens of five groups of mice. The precise treatment of each group is described in detail in Figure 2A. As expected, both the i.p. and the i.n. mCherry *Brucella* challenge was controlled well in mice chronically infected with wild-type *Brucella* (Figure 2B), thus confirming the presence of a protective memory response in these mice. In striking contrast, the CFU levels of wild-type *Brucella* were similar in unchallenged mice and in the i.p. and i.n. challenged groups (Figure 2C). This demonstrates that the memory response is able to eliminate the challenge strain but not the *Brucella* strain that has settled in the reservoir cells. To the best of our knowledge, this observation has never been reported before and suggests that the reservoir cells protect *Brucella* against the *Brucella*-specific memory response.

### *T. brucei* Infection Reduces the Number of *Brucella* Persisting in the Spleen

**Figure 3:**
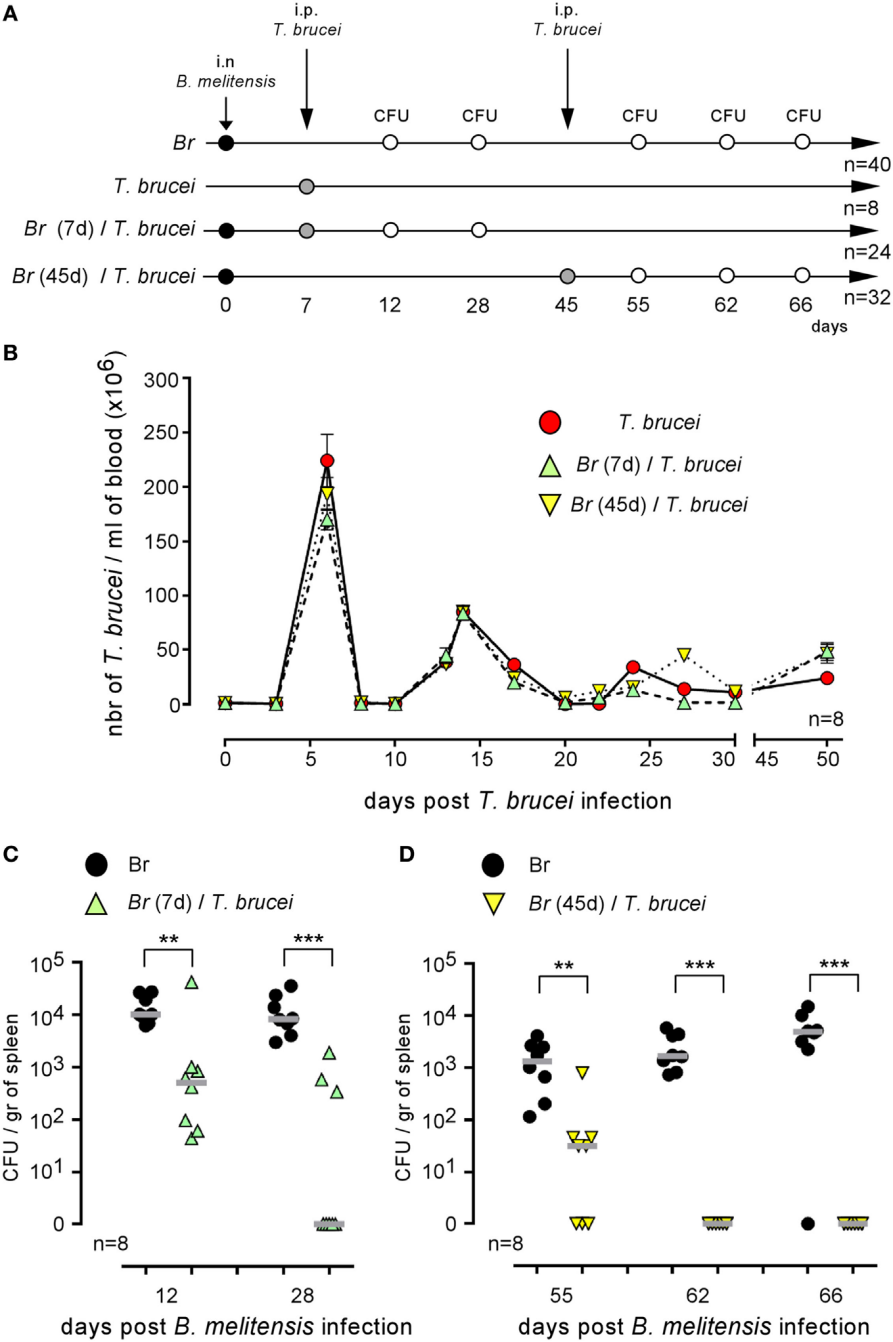
*Trypanosoma brucei* co-infection strongly reduced the CFU level of *Brucella melitensis* in the spleen. Wild-type C57BL/6 mice were infected i.n. with PBS or 2 × 10^4^ CFU of mCherry-*B. melitensis* and, at 7 or 45 days postinfection, received an i.p. injection of PBS or 5,000 *T. brucei* or PBS alone, as described in panel **(A)**. The data in panel **(B)** represent the number of *T. brucei* per milliliters of blood. The data in panels **(C,D)** represent the number of colony-forming units per gram of spleen. Note that, to avoid a possible effect of repeated blood sampling, mice that were bled to measure the number of parasites in the blood were not used to measure the number of *Brucella* CFUs in the spleen. Gray bars represent the median. *n* denotes the number of mice used for each group (**p < 0.01, ***p < 0.001). These results are representative of at least two independent experiments.

Co-infection could positively or negatively affect the control of ongoing infection [reviewed in Ref. (1)]. Wild-type C57BL/6 mice were infected i.n. with PBS or 2 × 10^4^ CFU of mCherry-*B. melitensis* and then received an i.p. injection of PBS or 5,000 parasites at 7 or 45 days post *Brucella* infection (as indicated in Figure 3A). At the selected time point following *T. brucei* infection, the number of parasites was measured in the blood (Figure 3B). No significant impact of *Brucella* infection on the course of *T. brucei* was detected. In the same experiment, at a selected time point following *Brucella* infection, the mice were sacrificed, the spleens were harvested, and the *Brucella* CFUs in the spleen were counted by plating (Figures 3C,D). Our results showed that both early (day 7, Figure 3C) and late (day 45, Figure 3D) infection with *T. brucei* induced a rapid decrease in the CFU count of *B. melitensis* in the spleen. At 5 days post *T. brucei* infection (Figure 3C), the CFU level was already decreased by >1 log in the co-infected mice compared to the mice infected with *B. melitensis* alone. Twenty-one days following early and late *T. brucei* infection (Figures 3C,D), the CFU level appeared to be reduced by approximately 3 log, and the *B. melitensis* CFU count was below the detection threshold (10 CFU/spleen) in the majority of the co-infected mice. Similar results were obtained in the i.p. model of *B. melitensis* infection (Figure S2 in Supplementary Material). These results suggest that the immune response induced by *T. brucei* infection is able to eliminate *Brucella* in its reservoir cells. It is important to remark that this effect is not due to depletion of the entire CD11c^+^ spleen cell population, like in CD11c-DTR mice treated with DT, as the frequency of CD11c^+^MHCII^+^ cells is not significantly reduced in the spleen of *T. brucei* co-infected mice compared to the spleen of *Brucella*-infected mice (Figure S3 in Supplementary Material).

**Figure 4:**
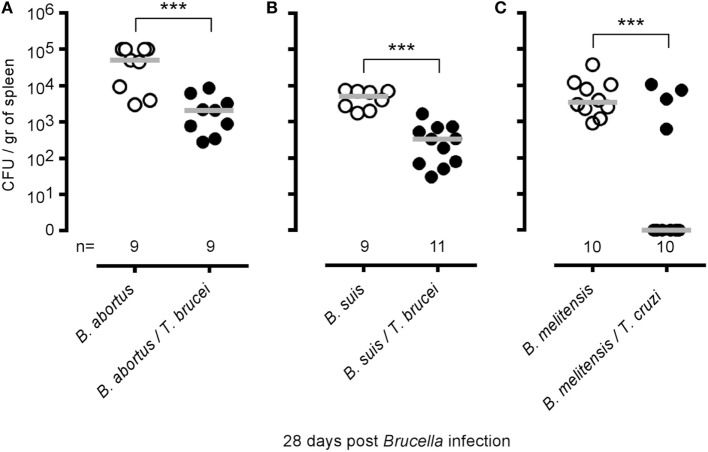
Impact of *Trypanosoma brucei* co-infection on *Brucella suis* and *Brucella abortus* infection and impact of *Trypanosoma cruzi* on *Brucella melitensis* infection. Wild-type C57BL/6 mice were infected i.n. with 2 × 10^4^ CFU of *B. abortus* **(A)** or *B. suis* **(B)** and, at 7 days postinfection, received an i.p. injection of 5,000 *T. brucei* in 200 µl of PBS or PBS alone. **(C)** Wild-type C57BL/6 mice were infected i.n. with 2 × 10^4^ CFU of mCherry-*B. melitensis* and, at 7 days postinfection, received an i.p. injection of 1,000 *T. cruzi* in 200 µl of PBS or PBS alone. The mice were euthanized at 28 days post *Brucella* infection, and the spleen was harvested. The data represent the number of colony-forming units per gram of spleen. Gray bars represent the median. *n* denotes the number of mice used for each group. These results are representative of at least two independent experiments (***p < 0.001).

To determine whether the elimination of *B. melitensis* observed after *T. brucei* infection could be the consequence of antigenic cross-reactivity, we tested whether *T. brucei* can affect the growth of two other *Brucella* species able to infect humans, *B. abortus* and *B. suis*, in the spleen (Figures 4A,B), and whether i.p. *T. cruzi* infection also reduces the level of *B. melitensis* (Figure 4C). *T. cruzi* is an intracellular parasite displaying different infection kinetics and does not share an antigen with *T. brucei*. We observed that *T. brucei* infection leads to a 10-fold reduction of *B. abortus* and *B. suis* CFUs in the spleen and that *T. cruzi* infection also induces a drastic reduction of *B. melitensis* in the spleen of infected mice. These results strongly suggest that elimination of *Brucella* following *Trypanosoma* infection is not due to shared antigens between the pathogens.

### Elimination of *Brucella* by *T. brucei* Requires Functional Il-12/IFNγ Signaling Pathways

**Figure 5:**
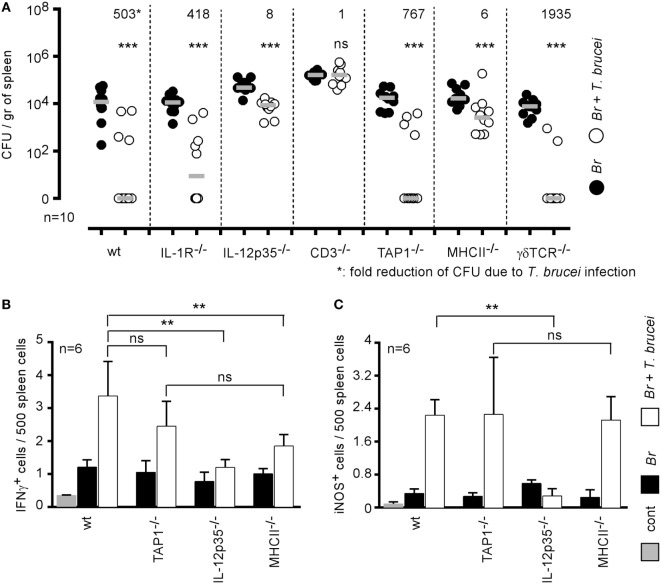
Elimination of *Brucella* during *Trypanosoma brucei* infection required IL-12 and CD4^+^ T cells. **(A)** Wild-type and various genetically deficient C57BL/6 mice were infected i.n. with 2 × 10^4^ CFU of mCherry-*Brucella melitensis* and, at 7 days postinfection, received an i.p. injection of 5,000 *T. brucei* in 200 µl of PBS (co-infection group) or PBS alone (*B. melitensis* group). The mice were euthanized at 28 days post *Brucella* infection, and the spleen was harvested **(A)**. The data represent the number of colony-forming units per gram of spleen. Gray bars represent the median. *n* denotes the number of mice used for each group. **(B,C)** The data represent the frequency of IFNγ- and iNOS-producing cells in the spleen as determined by flow cytometry analysis. The control (cont) group consisted of naive mice receiving PBS only (**p < 0.01, ***p < 0.001). These results are representative of at least two independent experiments.

IFNγ-producing CD4^+^ T cells are key players in the protective immune response against *B. melitensis* (16). When comparing the impact of *T. brucei* infection on *B. melitensis* in wild-type and various deficient mice, we observed that *T. brucei*-induced elimination of *B. melitensis* is strongly reduced in CD3^−/−^ (1-fold reduction of colony-forming unit), MHCII^−/−^ (6-fold), and IL-12p35^−/−^ mice (8-fold) but not in TAP1^−/−^ (767-fold), TCR-δ^−/−^ (1,935-fold), and IL1R^−/−^ mice (418-fold) (Figure 5A) when compared to wild-type mice (503-fold). These results suggest that the mechanism underlying the elimination of *B. melitensis* by *T. brucei* is indeed dependent on the host adaptive immune response and partially requires functional IL-12/IFNγ signaling pathways and CD4^+^ T cells. Elimination of *B. melitensis* following co-infection with *T. cruzi* is also dependent on IL-12 (Figure S2 in Supplementary Material). Note that depletion of CD4^+^ or CD8^+^ T cells by injection of depleting antibodies to confirm the data obtain with MHCII^−/−^ and TAP1^−/−^ mice is not feasible in this model. Indeed, CD11c^+^ cells constitute a potential *Brucella* reservoir cells, and it is well known that some subpopulations of splenic CD11c^+^ dendritic cells express CD4 or CD8 receptors.

As nitric oxide produced by iNOS/NOS2 is widely known to be produced in response to IFNγ in *B. melitensis* (34) and *T. brucei* (35) models and to negatively affect *B. melitensis* growth in the spleen (34), we analyzed by flow cytometry the frequency of IFNγ- and iNOS-producing cells in the spleen of wild-type and various deficient mice infected with *B. melitensis, T. brucei* or co-infected with both pathogens. Our results showed that *T. brucei* induced a strong increase in the frequency of IFNγ (Figure 5B) and iNOS-producing cells (Figure 5C) in the spleen. IFNγ is mainly produced by CD3^+^CD4^+^ T cells and iNOS is produced by CD11b^+^ cells (data not shown). As expected, the absence of IL-12p35 significantly reduces the frequency of IFNγ^+^ and iNOS^+^ cells. Interestingly, there was no significant difference between the frequency of IFNγ and iNOS-producing cells in the spleen of TAP1^−/−^ (deficient in CD8^+^ T cells) and MHCII^−/−^ (deficient in CD4^+^ T cells) mice, demonstrating that the absence of *T. brucei*-induced *B. melitensis* control in MHCII^−/−^ mice is indeed due to the absence of CD4^+^ T cells and not only to the lack of IL-12/IFNγ signaling pathways.

### *T. brucei* Infection Does Not Affect the Course of *M. tuberculosis* Infection in Mice

**Figure 6:**
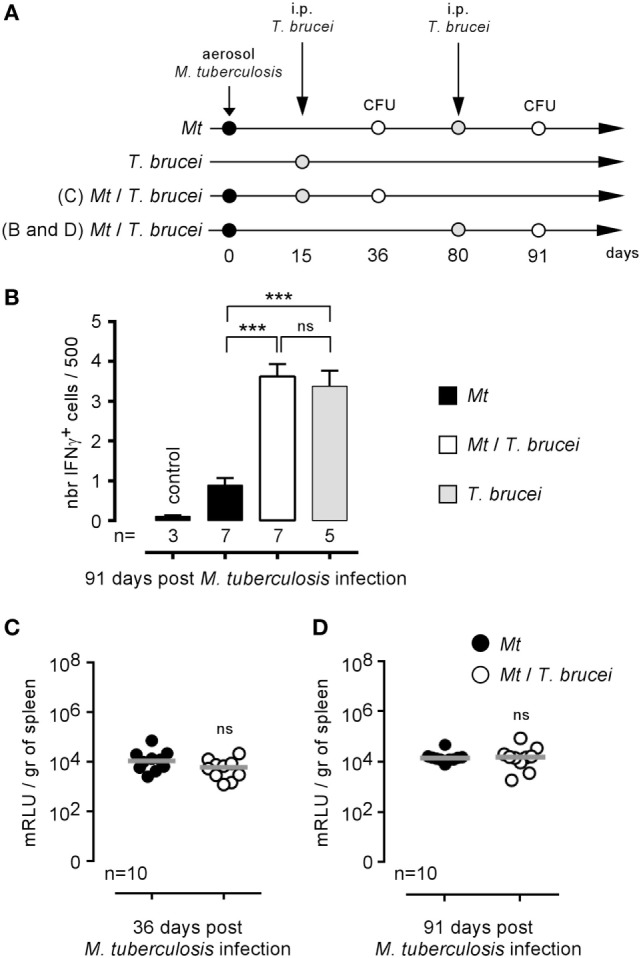
Impact of *Trypanosoma brucei* co-infection on the course of *Mycobacterium tuberculosis* infection in mice. Wild-type C57BL/6 mice were infected by aerosol with 5 × 10^3^ CFU of *M. tuberculosis* and, at 15 or 80 days postinfection, received an i.p. injection of 5,000 *T. brucei* in 200 µl of PBS (*M. tuberculosis*/*T. brucei* group) or PBS alone (*M. tuberculosis* group). The mice were euthanatized at the selected time post *M. tuberculosis* infection, and the spleen was harvested, as described in panels **(A,B)**. The data represent the frequency of IFNγ-producing cells in the spleen as determined by flow cytometry analysis. **(C,D)** The data represent the number of mRLU/g of spleen. Gray bars represent the median. *n* denotes the number of mice used for each group (***p < 0.001). These results are representative of at least two independent experiments.

*Brucella melitensis* and *M. tuberculosis* are both facultative intracellular bacteria able to chronically colonize the spleen of infected mice. It is clearly established that IFNγ production (36, 37) and activated CD4^+^ T cells (38) participate in the protective immune response against *M. tuberculosis* in mice, suggesting that bystander activation of these effector mechanisms by *T. brucei* could promote the control of *M. tuberculosis*. Thus, we tested the impact of i.p. *T. brucei* infection on the course of C57BL/6 mice previously infected by aerosol with *M. tuberculosis*, as described in Figure 6A. Surprisingly, we observed that, despite the enhanced frequency of IFNγ-producing cells in the spleen of co-infected mice (Figure 6B), *T. brucei* did not affect *M. tuberculosis* infection in the spleen either earlier (36 days, Figure 6C) or later (91 days, Figure 6D). This result demonstrates that *T. brucei* infection specifically impacts *B. melitensis* infection and that these effects cannot be generalized to all other intracellular bacterial infections.

### An Attenuated *T. brucei* Mutant Is Able to Reduce *Brucella* Infection

**Figure 7:**
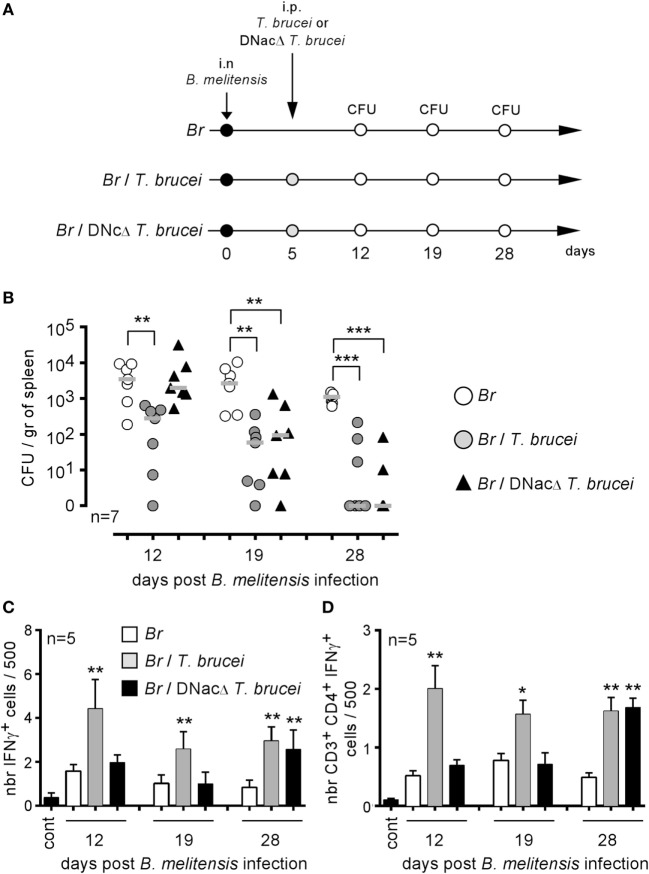
Attenuated dominant-negative adenylate cyclase (DNac) mutant of *Trypanosoma brucei* induced the elimination of *Brucella*. Wild-type C57BL/6 mice were infected i.n. with 2 × 10^4^ CFU of mCherry-*Brucella melitensis* and, at 7 days postinfection, received an i.p. injection of 5,000 wild-type *T. brucei* or attenuated DNac mutant of *T. brucei* in 200 µl of PBS or PBS alone. The mice were euthanized at 12, 19, and 28 days post *Brucella* infection and the spleen was harvested, as described in panel **(A,B)**. The data represent the number of colony-forming units per gram of spleen. Gray bars represent the median. *n* denotes the number of mice used for each group. **(C,D)** The data represent the mean of the frequency of IFNγ-producing cells **(C)** and CD3^+^CD4^+^ IFNγ-producing cells **(D)** in the spleen from five individual spleens as determined by flow cytometry analysis for each group. *n* denotes the number of mice used for each group (*p < 0.05, **p < 0.01, ***p < 0.001). These results are representative of at least two independent experiments.

Our previous experiments showed that *T. brucei* induces a strong inflammatory response mediated by IFNγ^+^CD4^+^ T cells in infected mice and that this response is indispensable to the reduction of *Brucella* infection in co-infected mice. To determine whether the intensity and duration of the IFNγ-mediated response are key parameters, we compared a wild-type *T. brucei* and a DNac *T. brucei* mutant (27), as described in Figure 7A. Infection with the DNac mutant induced a considerably lower parasitemia and a smaller inflammatory immune response (27). We observed that both wild-type and DNac *T. brucei* induced similar elimination of *B. melitensis* in co-infected mice (Figure 7B). The kinetics of elimination were very similar, except for the early time point of co-infection (day 12). The slower impact of the DNac mutant on *Brucella* could be due to the fact that it induces IFNγ less rapidly in spleen as demonstrated by the reduced frequency of IFNγ-producing cells in the spleen of DNac *T. brucei*-infected mice early in co-infection compared to wild-type *T. brucei*-infected mice (Figures 7C,D). Like for wild-type *T. brucei, Brucella* elimination by DNac *T. brucei* was impaired in IL-12p35^−/−^ mice (Figure S4 in Supplementary Material). On the whole, these results suggest that even lower persistence of parasite associated with lower levels of IFNγ is able to mediate *B. melitensis* elimination. However, live parasite seems to be required as we failed, in the same experiment, to reduce *B. melitensis* infection by three repeated injection (once per week) of a lysate of *T. brucei* (Figure S5 in Supplementary Material). The lysate has been realized, approximately, from a number of cultivated parasites corresponding to the number of parasites detected in the blood of infected mice at the peak of infection.

### Transient *T. brucei* Infection Does Not Alter Development of the Protective Memory Response against *B. melitensis*

*Trypanosoma brucei* infection has been described to deeply affect the protective humoral immune memory against unrelated pathogens (18). Thus, we planned to analyze the impact of *T. brucei* infection on the specific protective immune memory following *B. melitensis* infection. We have shown previously that humoral immunity-mediated control of secondary infection with *B. melitensis* is dependent on the route of infection. In the i.p. model, bacteria disseminate by the blood, and the humoral immune response is indispensable to efficiently control secondary infection (31). In contrast, bacteria disseminate slowly after i.n. infection, are undetectable in the blood, and the absence of B cells does not affect the control of secondary infection (16). We therefore chose to analyze the effects of *T. brucei* in both i.p. and i.n. *B. melitensis* infection models.

**Figure 8:**
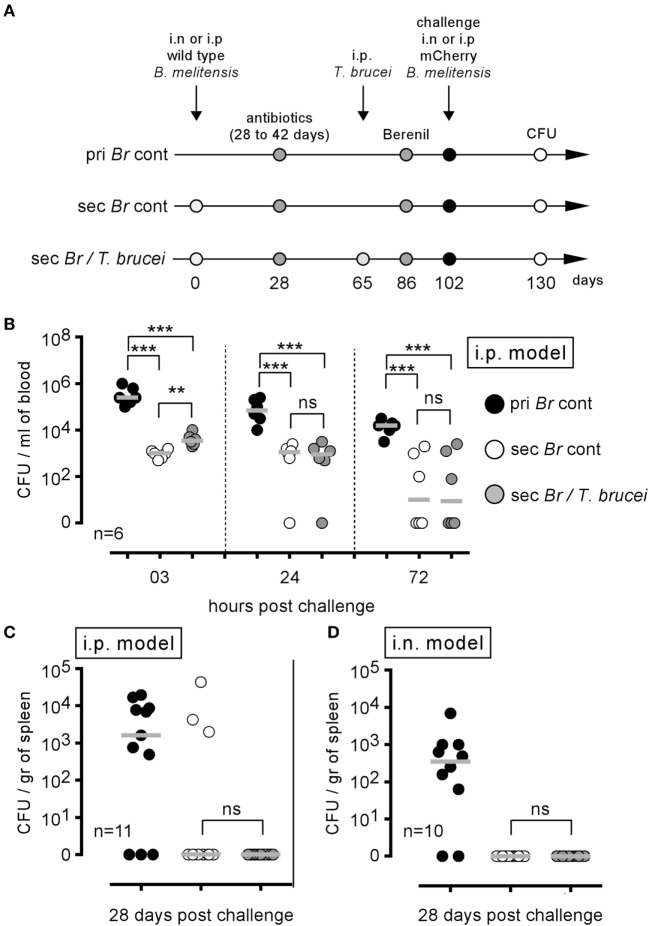
*Trypanosoma brucei* co-infection did not affect the protective memory against *Brucella melitensis*. As described in panel **(A)**, wild-type C57BL/6 mice were infected i.p. or i.n. with 2 × 10^4^ CFU of wild-type *B. melitensis* and treated with antibiotic at 28 days postinfection for 15 days. At 65 days, the mice received an i.p. injection of 5,000 *T. brucei* in 200 µl of PBS (Sec *Br*/*T. brucei* group) or PBS alone (Sec *Br* cont group) were treated with Berenil at 86 days and were challenged at 102 days with i.p. or i.n. injection of 2 × 10^4^ CFU of mCherry-*B. melitensis*. The pri *Br* cont group received only PBS, antibiotic, and Berenil until the *Brucella* challenge. **(B,C)** i.p. model: the data represent the number of colony-forming units per milliliter of blood or colony-forming units per gram of spleen at the selected time post challenge, as indicated. **(D)** i.n. model: the data represent the number of colony-forming units per gram of spleen. Gray bars represent the median. *n* denotes the number of mice used for each group (**p < 0.01, ***p < 0.001). These results are representative of at least two independent experiments.

Mice were i.p. infected with wild-type *B. melitensis* for 28 days, treated with antibiotic and then infected (Sec *Br*/*T. brucei* group) or not (Sec *Br* cont group) with *T. brucei*, as described in Figure 8A. Both groups of mice were treated with Berenil, an antiparasitic drug (28). After a resting period, the mice were i.p. challenged with mCherry *B. melitensis*. A group of naive mice, treated with antibiotic and Berenil, was also i.p. infected with mCherry *B. melitensis* and was used as the internal control (pri *Br* cont group). Surprisingly, *T. brucei* infection did not reduce the ability of *B. melitensis* immunized mice to control an i.p. *Brucella* challenge in the blood (Figure 8B) or the spleen (Figure 8C), despite a drastic reduction in the frequency of B cells (Figure S6A in Supplementary Material) and *Brucella*-specific IgM and IgG2a (Figures S6B,C in Supplementary Material). The same experiment in the *B. melitensis* i.n. infection model (i.n. infection and i.n. challenge with *B. melitensis*) gave similar results (Figure 8D). Thus, although *T. brucei* infection reduces the specific humoral response against *B. melitensis*, it does not impair the protective memory response controlling secondary *B. melitensis* infection in either the i.p. or i.n. infection model.

## Discussion

Immunology arose from the will and the need to ameliorate vaccination. During the 20th century, the molecular biology revolution led immunologists to develop highly reductionist experimental models, far removed from reality. For many years, the most studied immunological models were based on the injection of proteins combined with adjuvants. Much of our understanding of how the immune system works derives from studies of these models. We must not forget that our perception of reality results from the experimental model in which we study it. It may be time to remember that the first successful vaccination actually derives from a cross-reaction between smallpox and the cowpox virus and that multiple unrelated infections in the same host are probably more common than single mono-species infections. The impact of past or chronic unrelated infections on the ability of the host to control infection is well documented [reviewed in Ref. (1)], but the mechanisms remain largely unknown. In this study, we developed an original experimental model of co-infection to study the impact of *T. brucei* infection on the course of chronic infection with *B. melitensis* in mice.

Over the course of evolution, *Brucella* has acquired specific stealth strategies that allow it to interfere with its recognition by the immune system and neutralize immune effector mechanisms. For example, after phagocytosis, *Brucella* controls the intracellular trafficking of its vacuole to avoid degradation by phagolysosomes [reviewed in Ref. (39)]. We have shown previously in a mice model that following i.p. (15) or i.n. infection (16) the spleen is stably and durably colonized by *Brucella* and constitutes one of the major reservoirs of the infection. In the spleen of highly susceptible IL-12p40^−/−^ BALB/c mice, *Brucella* persists within specific CD11c^+^CD205^+^arginase1^+^ myeloid cells displaying high levels of lipids (32). In this study using CD11c-DTR C57BL/6 mice, we demonstrated that specific elimination of CD11c^+^ cells reduced colony-forming unit counts of *Brucella* in the spleen, suggesting that *Brucella* survives in CD11c^+^ reservoir cells in resistant C57BL/6 mice. Our results showed that these reservoir cells constitute a niche that hides *Brucella* from the primary immune response and even against the protective *Brucella*-specific memory response. Indeed, in mice chronically infected since 45 days with wild-type *B. melitensis*, a new infection with mCherry *B. melitensis* challenge strain is completely neutralized. But this specific memory response appears to be unable to eradicate the established wild-type *Brucella* strain. Thus, during chronic infection phase, *Brucella* appears to be extremely well equipped to escape the IFNγ-mediated protective immune response (16) and persist in the host. It is therefore really surprising and unexpected, in view of the well documented immunosuppressive effects of many trypanosomes (40), that both *T. brucei* and *T. cruzi* infection lead to an almost 2–3 log reduction of the *B. melitensis* load in the spleen of co-infected mice. *T. brucei* is known to suppress both the T-cell (19, 20) and B-cell (18) response in mice. *T. cruzi* infection reduces the humoral immune response against sheep erythrocytes in humans (41). To the best of our knowledge, neither *T. brucei* nor *T. cruzi* infection has been previously associated with an increased response to unrelated pathogens. A study by Lowry et al. (42) compared the course of *B. abortus* S19 in control and *Trypanosoma musculi*-infected mice. In striking contrast with our results, Lowry et al. showed that *T. musculi* favor *B. abortus* infection by suppressing the IFNγ response. The difference between Lowry’s co-infection model and our model may be due to the different timing of co-infection and *Trypanosoma* species used. Especially, *T. brucei* and *T. cruzi* induce a greater inflammatory response in mice than does *T. musculi*.

The fact that two species of *Trypanosoma* displaying completely different antigens, infectious cycles and dynamics are both able to favor the elimination of three different species of *Brucella* (*melitensis, suis*, and *abortus*) suggests that the underlying mechanism has very little chance of being based on antigenic cross-reactivity. The comparison of various deficient mice has demonstrated that CD4^+^ T cells, but not CD8^+^ T cells or γδ^+^ T cells, are indispensable to *Brucella* elimination, and thus that the immune response is directly implicated in this phenomenon. We also observed that functional IL-12p35/IFNγ signaling pathways are required. Taken together, these results suggest that the strong inflammatory IFNγ-mediated response induced by *T. brucei* and *T. cruzi* infection is responsible for the elimination of *Brucella*. Interestingly, in the absence of CD4^+^ T cells (MHCII^−/−^ mice), *T. brucei* induced a strong IFNγ response mediated by CD8^+^ T cells but was not able to reduce the level of *Brucella* persistence in the spleen, thus suggesting that IFNγ production is necessary but not sufficient to eliminate *Brucella* and that CD4^+^ T cells are key actors in this process. Infection with a strongly attenuated *T. brucei* mutant, but not repeated injection of a *T. brucei* lysate, was able to eliminate *Brucella* in the spleen, suggesting that the level of inflammation is not a limiting factor but that a living parasite is required. B cell deficient mice have been shown to display enhanced control of *Brucella* infection (43), and B cells have been reported to act as reservoir cells for *Brucella* in the spleen (44). As *T. brucei* is well known to induce B cell apoptosis (18), we cannot exclude that *Brucella* elimination by *T. brucei* could in part be a consequence of B cell depletion by *T. brucei*. However, *T. brucei* infection does not completely eliminate B cells in our model (Figure S5 in Supplementary Material). In addition, the colony-forming unit count reduction induced by *T. brucei* is already observed after 5 days, while no impact on the frequency of B cells in the spleen is detected at the same time [data not shown and Ref. (18)].

The fact that *T. brucei* infection affects *B. melitensis, B. suis*, and *B. abortus*, but not early or late phases of *M. tuberculosis* infection in the spleen, demonstrates that the mechanism implicated is specific to *Brucella* species but is not effective against all intracellular bacterial infections. However, the results obtained with *M. tuberculosis* are unexpected given the strong available evidence that a deficiency of IFNγ responses is associated with increased susceptibility to *M. tuberculosis* and non-tuberculous mycobacterial infections in animal models and humans (45, 46). In addition, Sakai et al. (47) recently nicely confirmed the protective role of IFNγ-producing CD4^+^ T in the control of extra-pulmonary *M. tuberculosis* infection. In our experimental model, we observed decreased IFNγ production *in vitro* in response to known MHC class II epitopes of *M. tuberculosis* antigens in splenocytes isolated from mice co-infected with *M. tuberculosis* and *T. brucei* compared to the responses measured in splenocytes from mice infected with *M. tuberculosis* (data not shown). This suggests that co-infection with *T. brucei* could have a negative impact on IFNγ responses by *M. tuberculosis* specific CD4^+^ T cells and that direct recognition of infected cells by specific CD4^+^ T cells rather than the presence of high levels of IFNγ may be essential to restricting the growth of *M. tuberculosis*. In addition, we cannot exclude that the beneficial impact of IFNγ production may be counterbalanced by some immunosuppressive effects associated to *T. brucei* infections.

Finally, as *T. brucei* is especially known for its ability to suppress vaccine-induced protective humoral memory (18), we tested the ability of *T. brucei* to neutralize *Brucella*-induced protective memory in i.p. and i.n. infection models. As expected, we observed a significant decrease in the B cell count in the spleen and a reduction of *Brucella*-specific IgM and IgG2a levels in the serum of co-infected mice compared to control *Brucella*-infected mice. Interestingly, similar results have been reported in cattle infected with either *Trypanosoma congolense* or *Trypanosoma vivax* that display suppressed humoral immune responses to attenuated *B. abortus* injected subcutaneously (48). Despite this reduction of the *Brucella*-specific humoral response, we did not observe a reduced ability of the humoral response to neutralize *Brucella* in the blood or to impair *Brucella* persistence in the spleen.

On the whole, our results show that both *T. brucei* and *T. cruzi* infection are able to positively improve the immune control of *Brucella* infection in the spleen. This demonstrates that non-antigen specific effector mechanisms could be more efficient at eliminating stealth pathogens like *Brucella* than antigen specific immune effectors. This finding opens up new perspectives for research on brucellosis as well as for other stealth pathogens. This co-infection model also offers a unique opportunity to identify the effector mechanisms expressed by CD4^+^ T cells involved in the elimination of *Brucella* reservoir cells and could be further used to discover new therapeutic strategies for brucellosis without antibiotic treatment. As mice are not a natural host for *B. melitensis* and *T. brucei*, extrapolation of these results to natural hosts must be done carefully. However, the geographic distribution of these pathogens is largely overlapping, and all of them infect cattle and humans. Thus, co-infection of the natural host with *Brucella* and *Trypanosoma*, although not documented, should not be uncommon, and these effects deserve to be studied in nature.

## Ethics Statement

The procedures used in this study and the handling of the mice complied with current European legislation (directive 86/609/EEC) and the corresponding Belgian law “Arrêté royal relatif à la protection des animaux d’expérience du 6 avril 2010 publié le 14 mai 2010.” The Animal Welfare Committee of the Université de Namur (UNamur, Belgium) reviewed and approved the complete protocol (Permit Number: 12-188).

## Author Contributions

EM wrote the article. AM, MV, GP, AD, and HT performed the experiments. CDT, GV, MR, CT, and J-JL provided biological materials.

## Conflict of Interest Statement

The authors declare that the research was conducted in the absence of any commercial or financial relationships that could be construed as a potential conflict of interest.
