# Supplementary figures and images for: Trypanosoma Infection Favors Brucella Elimination via IL-12/IFNγ-Dependent Pathways
Source: Front Immunol. 2017 Jul 31;8:903. doi: 10.3389/fimmu.2017.00903 (PMC5534484; doi:10.3389/fimmu.2017.00903)

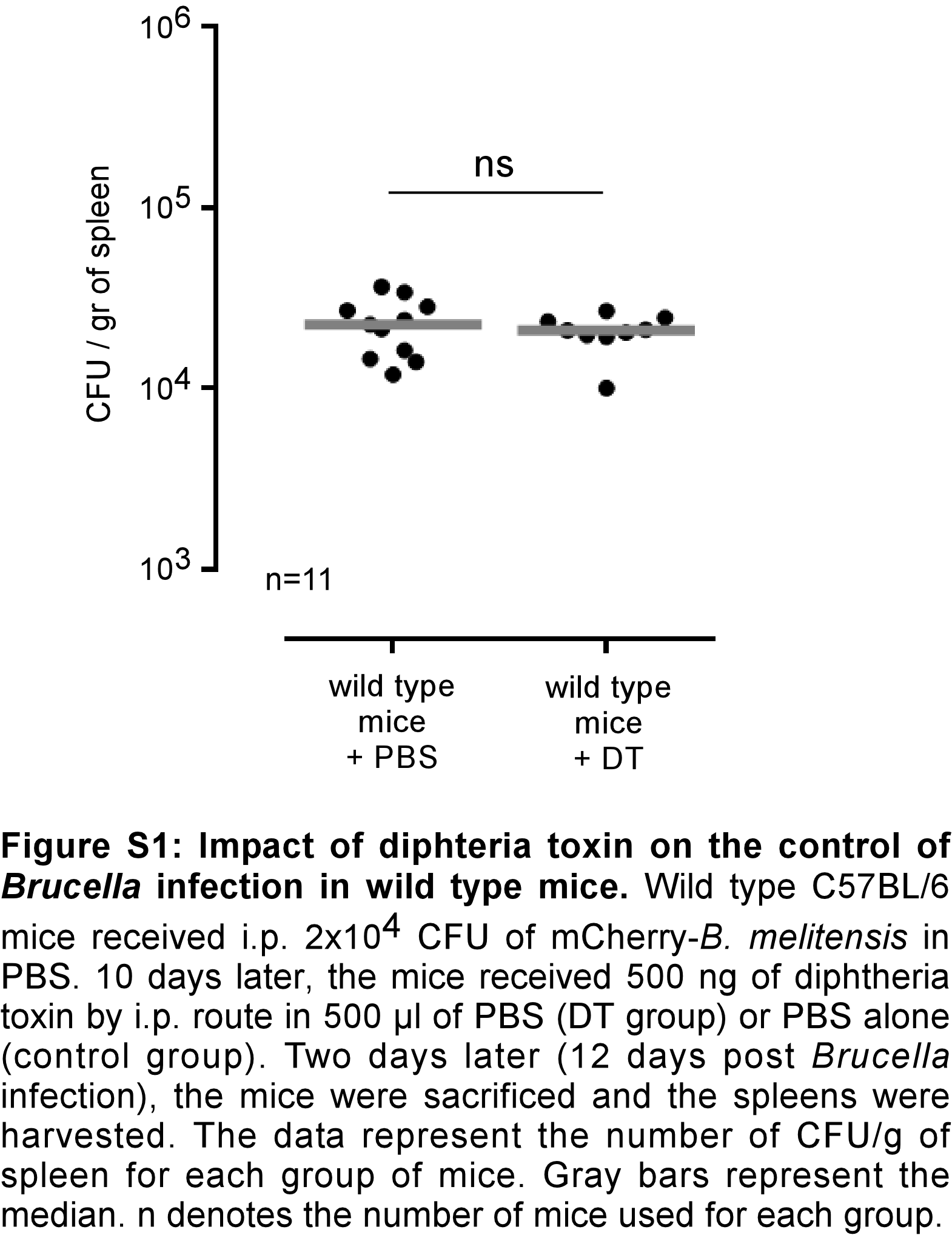

Supplement: Supplementary file 1 [file image_1.tif]

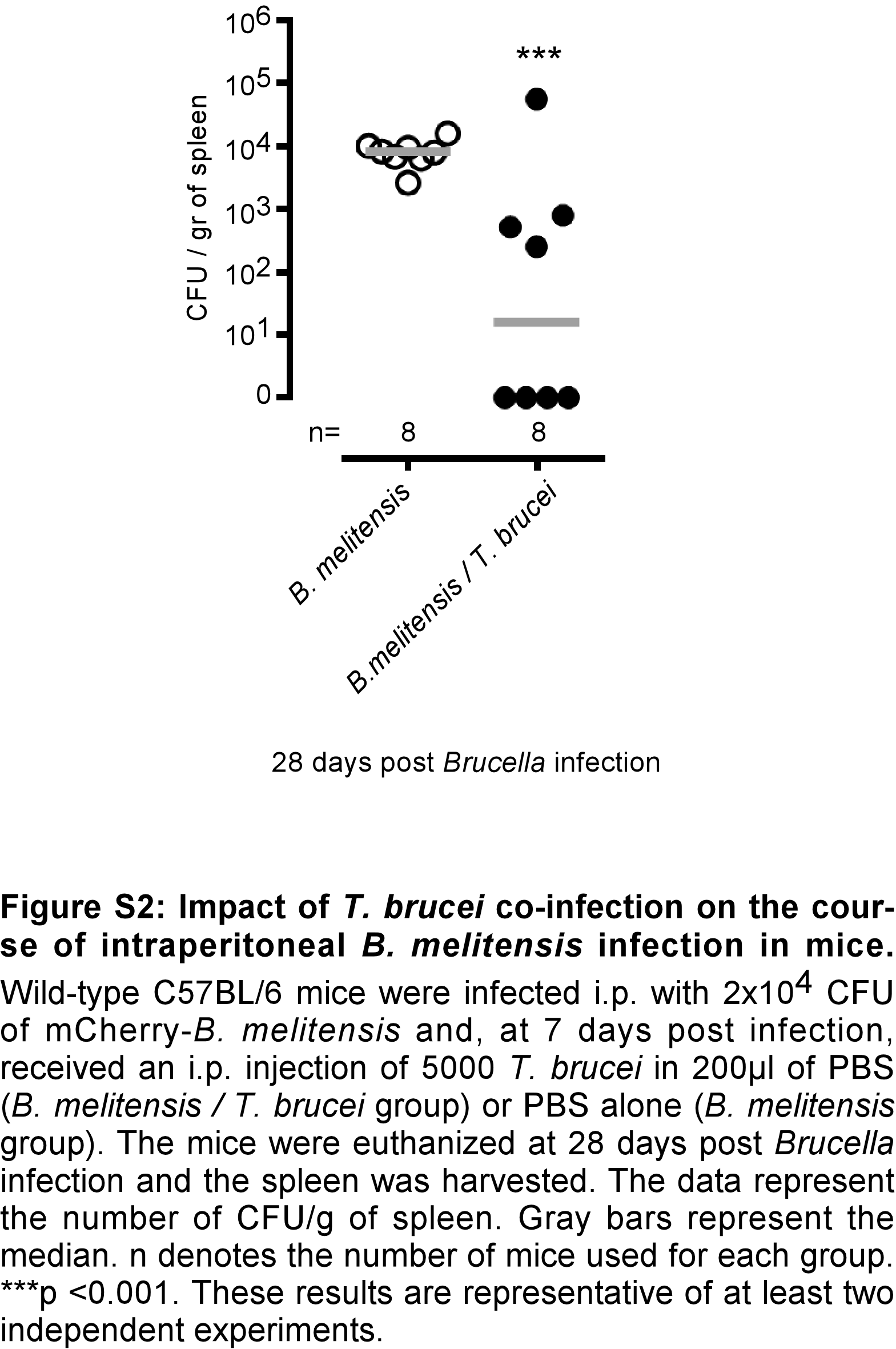

Supplement: Supplementary file 2 [file image_2.tif]

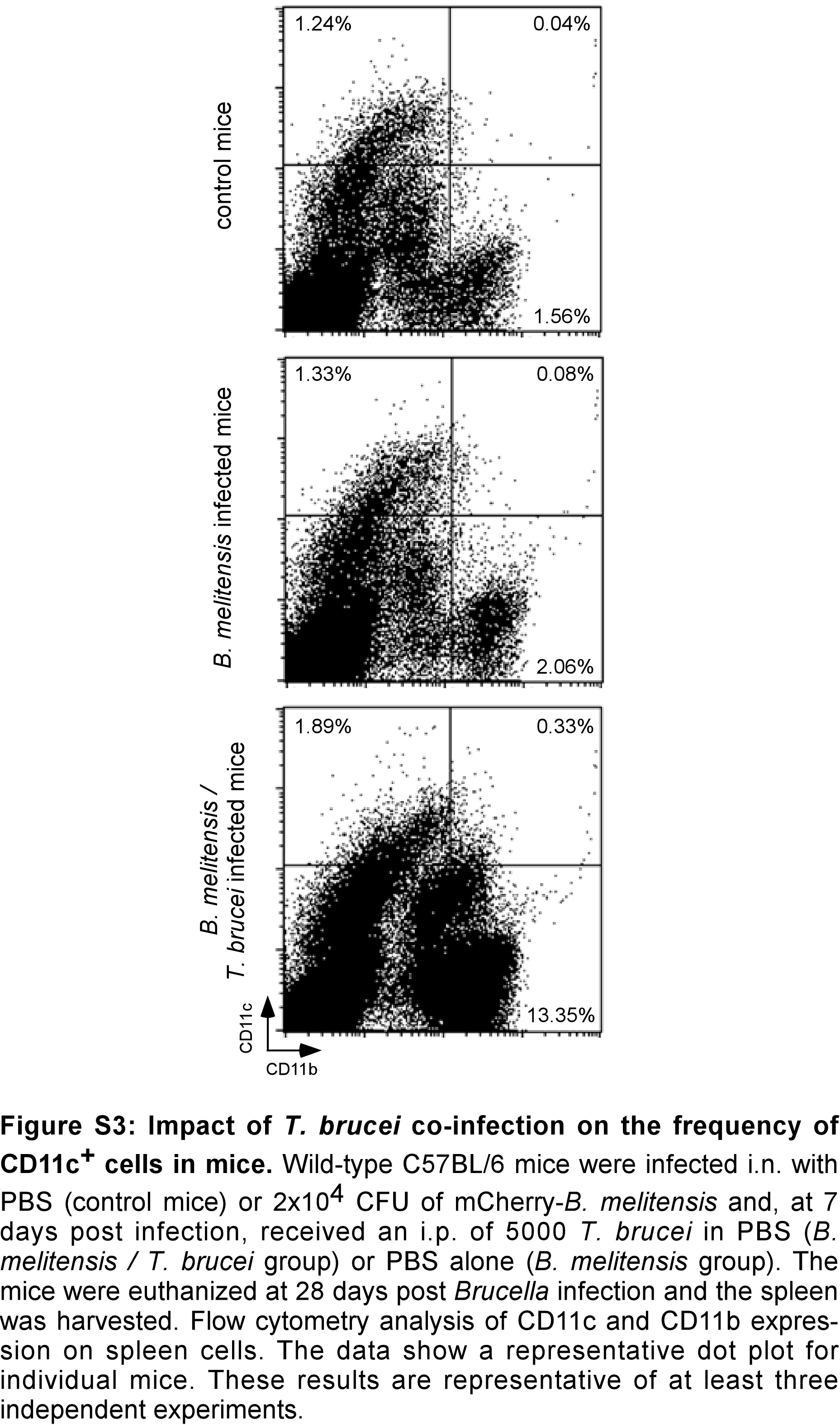

Supplement: Supplementary file 3 [file image_3.tif]

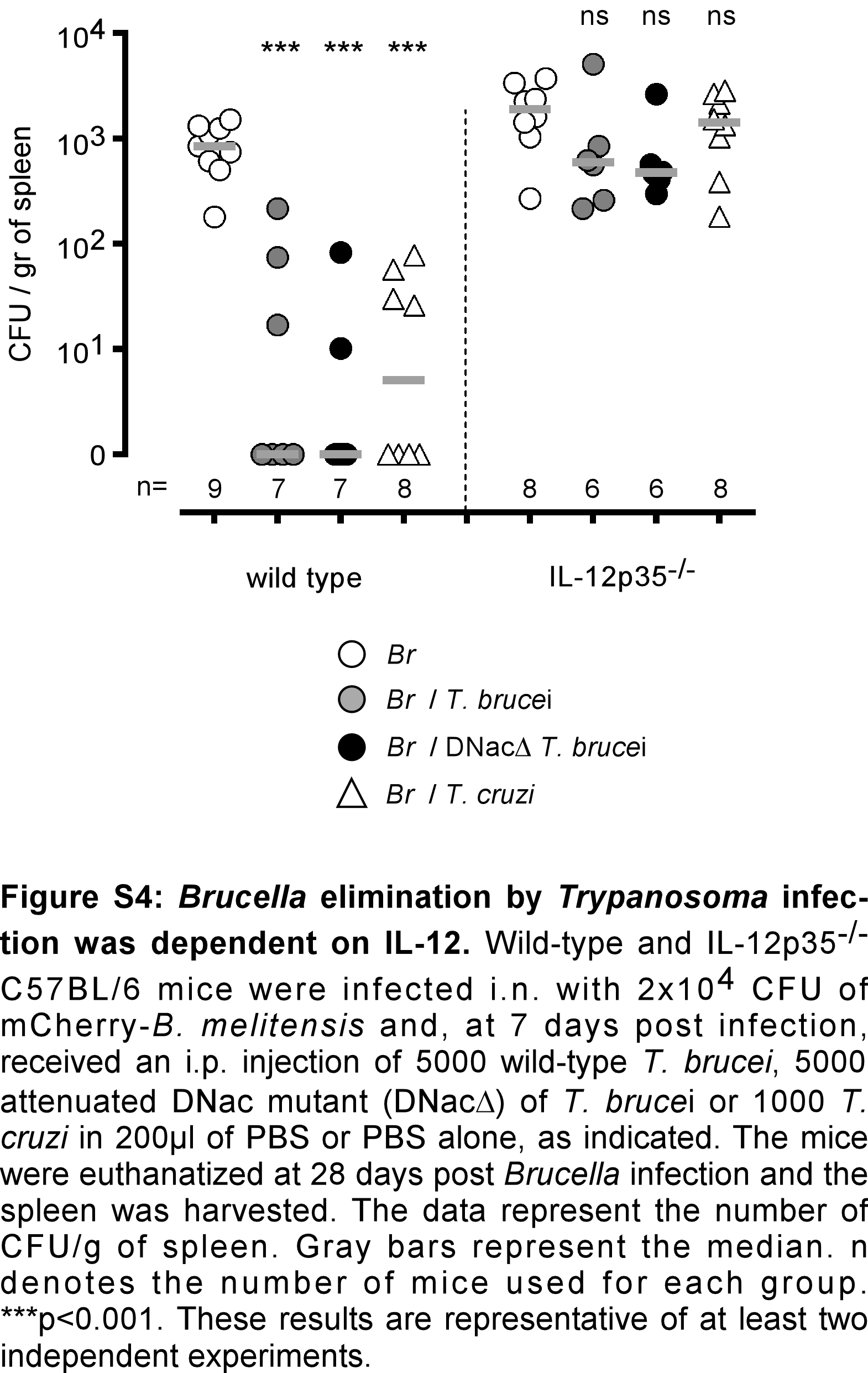

Supplement: Supplementary file 4 [file image_4.tif]

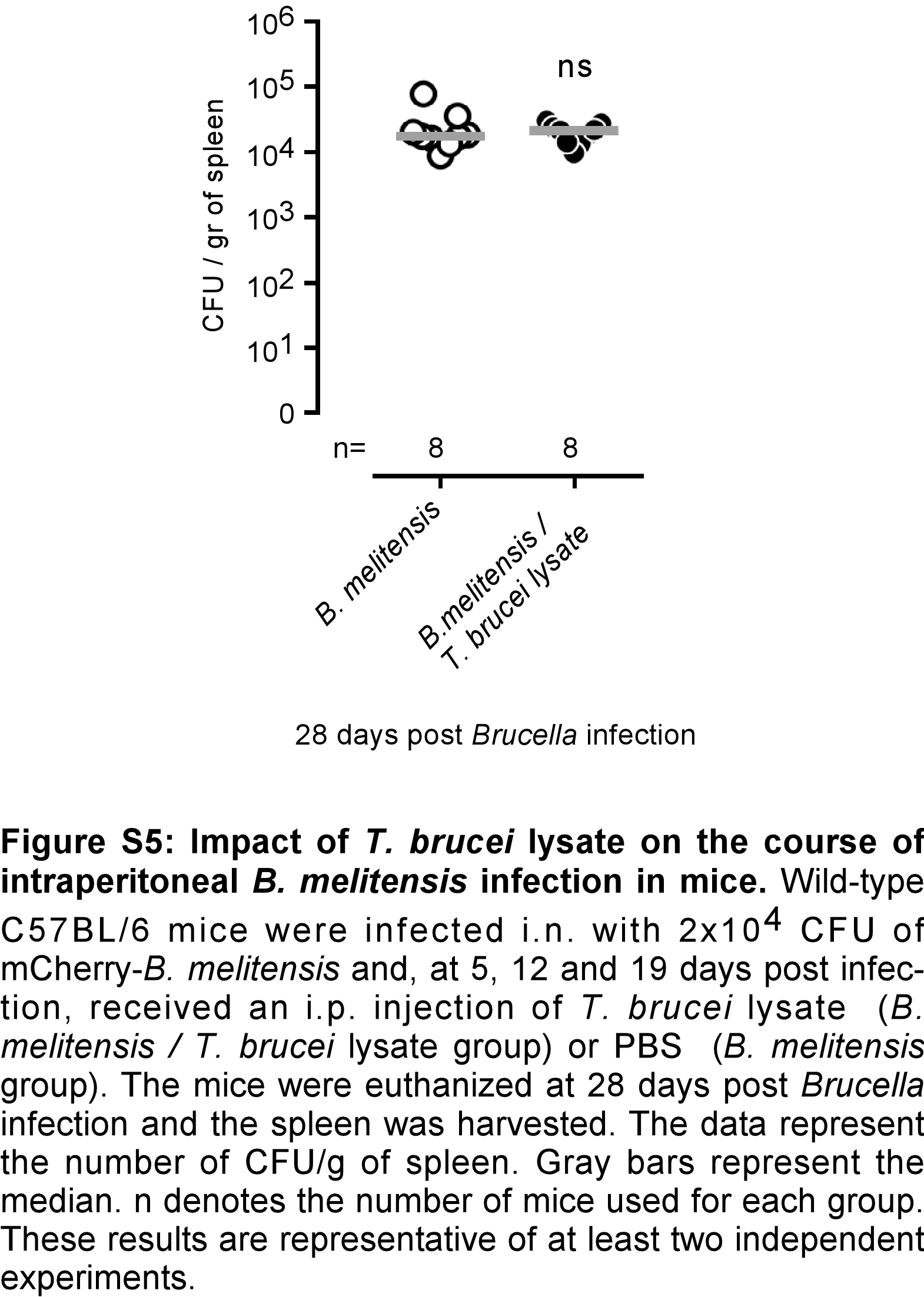

Supplement: Supplementary file 5 [file image_5.tif]

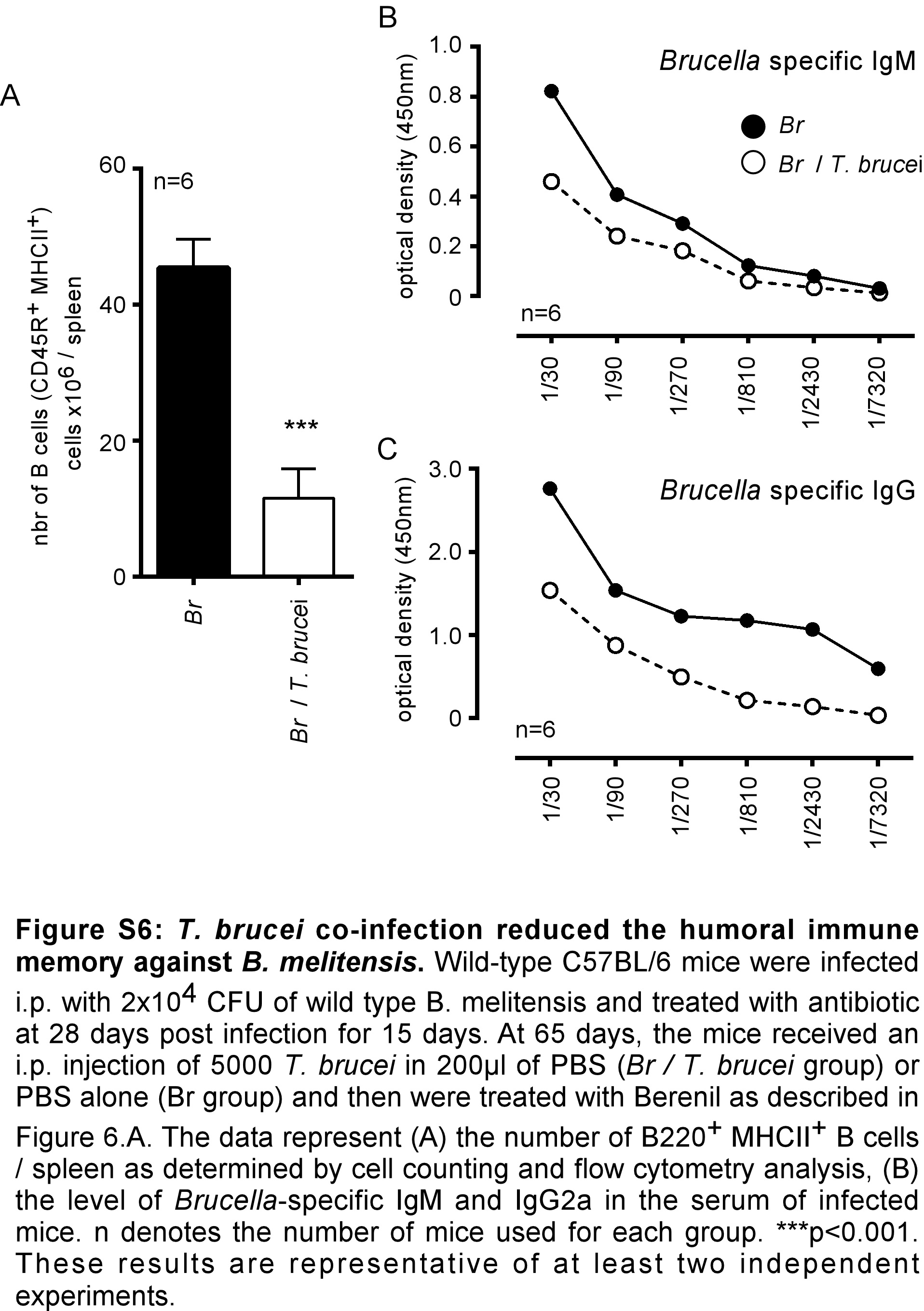

Supplement: Supplementary file 6 [file image_6.tif]
